# Evaluation of the SAMe-TT_2_R_2_ score to predict the quality of anticoagulation control in patients after mitral valve replacement

**DOI:** 10.3389/fsurg.2025.1561690

**Published:** 2025-06-09

**Authors:** Yilin Li, Yang Gao, Qiuming Hu, Xu Meng, Shubin Li

**Affiliations:** ^1^ Zhongshan Medical College, Sun Yat sen University, Guangzhou, China; ^2^ Department of Cardiology Surgery, Dalian Municipal Central Hospital, Dalian, China; ^3^ Department of Cardiology Surgery, Beijing Anzhen Hospital, Capital Medical University, Beijing, China; ^4^ Department of Cardiology Surgery, Xinxiang Central Hospital, The Fourth Clinical College of Xinxiang Medical College, Xinxiang, China

**Keywords:** SAMe-TT_2_R_2_ score, anticoagulation control, time in therapeutic range, mitral valve replacement, TTR

## Abstract

**Background:**

This study aimed to evaluate the role of the SAMe-TT_2_R_2_ score in the prediction of anticoagulation control after mechanical mitral valve replacement.

**Methods and results:**

We retrospectively reviewed clinical data of 160 patients who received mechanical mitral valve replacement at Beijing Anzhen Hospital from January to December 2013. Collected data included the patient's general information and any history of medication, smoking, post-operative embolism due to anticoagulant, bleeding complications, and death information. In the SAMe-TT_2_R_2_ score results, the lowest score was 2 points (5.6%), and the highest score was 7 points (0.6%). The number of people with 4 points was the largest (69 people, 43.1%). When the cut-off value of the SAMe-TT_2_R_2_ score was set to ≥4, the sensitivity and specificity of predicting Time in Therapeutic Range (TTR) ≥65% were 69.8% and 93.1%, respectively. The Youden index was 0.629. If the cut-off value of the SAMe-TT_2_R_2_ score was set to ≤4, the sensitivity and specificity of predicting TTR ≥65% were 93.0% and 44.1%, respectively, and the Youden index was 0.371. The Receiver Operator Characteristic (ROC) curve evaluates the predictive power of the SAMe-TT_2_R_2_ score for TTR ≥65%. The figure showed that when the cut-off point was ≥4, the best combination of sensitivity and specificity was shown (69.8% and 93.1%, respectively). The Area Under the Curve (AUC) was 0.854.

**Conclusions:**

After mechanical mitral valve replacement, the SAMe-TT_2_R_2_ model can effectively predict the TTR level during the course of oral warfarin anticoagulation therapy. The SAMe-TT_2_R_2_ score ≥4 can predict TTR <65%.

## Introduction

Thrombotic complications are prone to occur after mechanical mitral valve replacement, and patients need long-term oral warfarin anticoagulation therapy (1). The complications, such as thromboembolism and bleeding caused by improper anticoagulation, seriously affect the patient's long-term life quality and prognosis after the surgery (2). During the postoperative follow-up of these patients, their anticoagulant level will be closely monitored by doctors. Time in Therapeutic Range (TTR) is an important indicator of the level of anticoagulation quality. A high TTR score indicates that the patient's anticoagulation situation is good with a low probability of anticoagulation-related complications (3). How to use a simple evaluation method to let doctors quickly understand the TTR level of patients is very useful for busy clinical work. This study found that the SAMe-TT_2_R_2_ score (**S**: gender, **A**: age, **Me**: medical history, **T**: treatment, **T**: smoking, **R**: Race,) can effectively predict the level of TTR, which provides a reference for further improving the quality of postoperative anticoagulation.

## Materials and method

This was an observational retrospective cohort study conducted among 168 patients who underwent mechanical mitral valve replacement at Beijing Anzhen Hospital from January to December 2013. None of the patients had any previous history of bleeding disorders and consented to take warfarin for anticoagulation therapy after the operation. During the operation, tricuspid valve repair or atrial fibrillation radiofrequency ablation can be performed simultaneously. Patients (3, 1.8%) who couldn't adhere to the warfarin treatment post-surgery were excluded. We also excluded patients with severe liver and kidney dysfunction (1, 0.6%), hyperthyroidism (1, 0.6%), those who had a coronary artery bypass grafting and aortic valve replacement during the operation (1, 0.6%), pregnant patients (1, 0.6%), as well as patients unable to complete the follow-up (1, 0.6%). A total of 160 cases were included in this study, and the loss to follow-up rate was 4.8%.

Collected data included patients' general information and history of medication, smoking, post-operative embolism due to anticoagulant, bleeding complications, and death information. We collected the information through telephone follow-up, outpatient review, WeChat, and the database of the Beijing Anzhen Hospital. During the follow-up period, the patients' International Normalized Ratio (INR) values were recorded, and the INR cycle was monitored. In 2017, the European Society of Cardiology (ESC) and the European Society of Cardiothoracic Surgery (EACTS) released the guidelines for managing valvular heart disease. They recommended a target value of 3.0 for the INR (4). However, the biological differences between races and regions have caused significant differences in anticoagulant strength. In Asia, low-dose anticoagulation is highly recommended (5–8). Based on previous research and practice in our heart center, we determined the target value of INR to be 1.8–2.2.

TTR calculation: TTR is a way to evaluate the quality of anticoagulation control. A higher TTR score can effectively reduce warfarin-related bleeding and thrombosis. The Rosendaal method (using the time redistribution between INR of two adjacent tests) was used to calculate TTR (9). The anticoagulation quality is satisfactory when the TTR score is ≥65% (10, 11).

SAMe-TT_2_R_2_ score definition: **S**: gender, 1 point for female; **A**: age, 1 point for <60 years old; **Me**: medical history, 1 point for people with more than 2 comorbidities, including hypertension, diabetes, coronary artery disease, myocardial infarction, peripheral artery disease, congestive heart failure, history of stroke, lung disease, and liver or kidney disease; **T**: treatment, 1 point, such as amiodarone for rhythm control; **T**: smoking (within 2 years), 2 Points; **R**: Race, 2 points for non-white (12). As all the patients in our study are Chinese, the minimum score is 2 points.

## Statistical analysis

Measurement data were expressed as mean ± standard deviation (х ± s), performed by normality test. We compared categorical data using the chi-square test. All tests were two-sided, and *p*-values <0.05 were considered significant. Calculations were performed using SPSS software (version 22.0). The ROC (Receiver Operator Characteristic) curve was used to determine the best node and predict the performance of sub-optimal TTR. The Hosmer-Lemeshow test was used to verify the accuracy of the model prediction probabilities, and SPSS22.0 was used to calculate the Hosmer-Lemeshow test for this model. When *P* > 0.05, the model prediction probabilities were consistent with actual observations. Kaplan–Meier survival curve was used to analyze the death of patients with different SAMe-TT_2_R_2_ scores. Patients were divided into 2 groups according to SAMe-TT_2_R_2_ score ≥4 and SAMe-TT_2_R_2_ score ≤4. Chi-square test was used to analyze whether there was statistical difference between the two groups.

## Results

160 patients completed the follow-up research (54 males, 106 females, mean age 53.4 ± 8.4 years), and the median follow-up time was 1,749 (1,631, 2,004) days. In the SAMe-TT_2_R_2_ score results, the lowest score was 2 points (5.6%) and the highest score was 7 points (0.6%). The number of people with 4 points was the largest (69 people, 43.1%). The demographic characteristics of the samples are shown in Table 1.

**Table 1:** Demographic characteristics of the sample.

Variable	*n* = 160
Female sex	106 (66.3)
Age (years, mean ± SD)	53.4 ± 8.4
Hypertension	38 (23.8)
Diabetes	44 (27.5)
Coronary heart disease	46 (28.8)
Peripheral vascular disease	2 (1.3)
Stroke	17 (10.6)
Heart failure	22 (13.8)
Lung disease	13 (8.1)
Liver and kidney disease	7 (4.4)
SAMe-TT_2_R_2_ score	
2, *n* (%)	9 (5.6)
3, *n* (%)	32 (20.0)
4, *n* (%)	69 (43.1)
5, *n* (%)	42 (26.3)
6, *n* (%)	7 (4.4)
7, *n* (%)	1 (0.6)

Categorical variables are shown as *n* (%), and continuous variables, as median (25%–75%).

During the follow-up period, a total of 4,087 INR examinations were performed, and the median INR was 2.07, Mean ± SD is 2.05 ± 0.55, interquartile range (IQR) is 0.50.Of the INR measurements, 2,207 (54.0%) were within the target range (1.8–2.2), 1,102 (27.0%) were supratherapeutic (>2.2), and 778 (19.0%) were subtherapeutic (<1.8). The median TTR was 43.8% (IQR 31.7%–65.6%).

Among the various indicators used for evaluating the SAMe-TT_2_R_2_ score, apart from ethnic factors, gender factors were the most common (106, 66.3%). In contrast, the use of amiodarone for rhythm control and smoking within 2 years were the least common (30, 18.6%) (see Table 2).

**Table 2:** Prevalence of the SAMe-TT_2_R_2_ score components.

Score component		*n* (%)
S	Sex (female)	106 (66.3)
A	Age (<60 years)	76 (47.5)
Me	Medical history (>2 comorbidities^a^)	70 (43.8)
T	Treatment (amiodarone)	30 (18.8)
T_2_	Tobacco use (within 2 years)	30 (18.8)
R_2_	Race (non-Caucasian)	160 (100)

^a^ Hypertension, diabetes, coronary artery disease/myocardial infarction, peripheral artery disease, previous stroke, congestive heart failure, lung disease, liver or kidney disease.

When the cut-off value of the SAMe-TT_2_R_2_ score was set to ≥4, the sensitivity and specificity of predicting TTR ≥65% were 69.8% and 93.1%, respectively. The Youden index was 0.629. If the cut-off value of SAMe-TT_2_R_2_ score was set to ≤4, the sensitivity and specificity of predicting TTR ≥65% were 93.0% and 44.1%, respectively, and the Youden index was 0.371.

**Figure 1:**
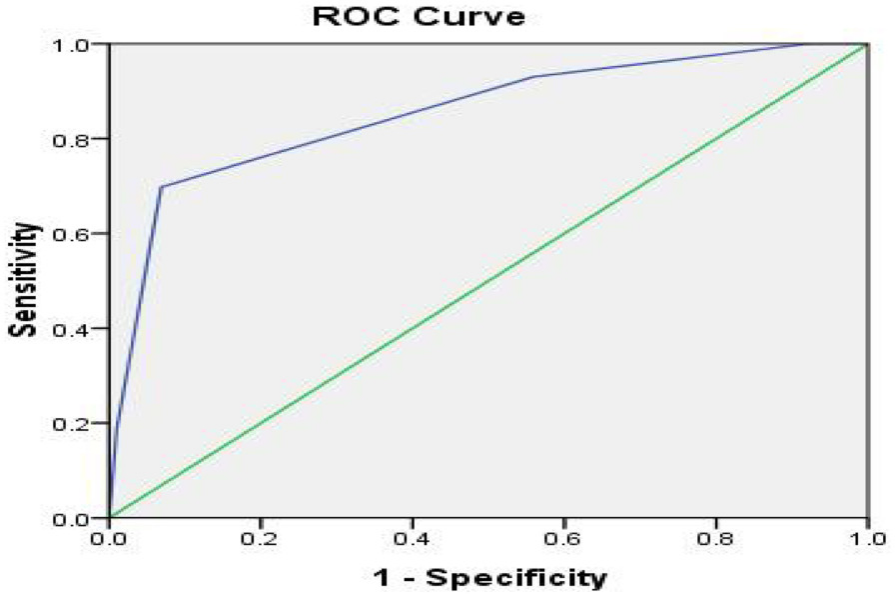
ROC curve for the outcome TTR ≥65%.

ROC curve (Figure 1) evaluates the predictive power of the SAMe-TT_2_R_2_ score for TTR ≥65%. The figure showed that when the cut-off point was ≥4, the best combination of sensitivity and specificity was shown (69.8% and 93.1%, respectively). The AUC was 0.854. The result of Hosmer-Lemeshow test is *P* = 0.713.

**Figure 2:**
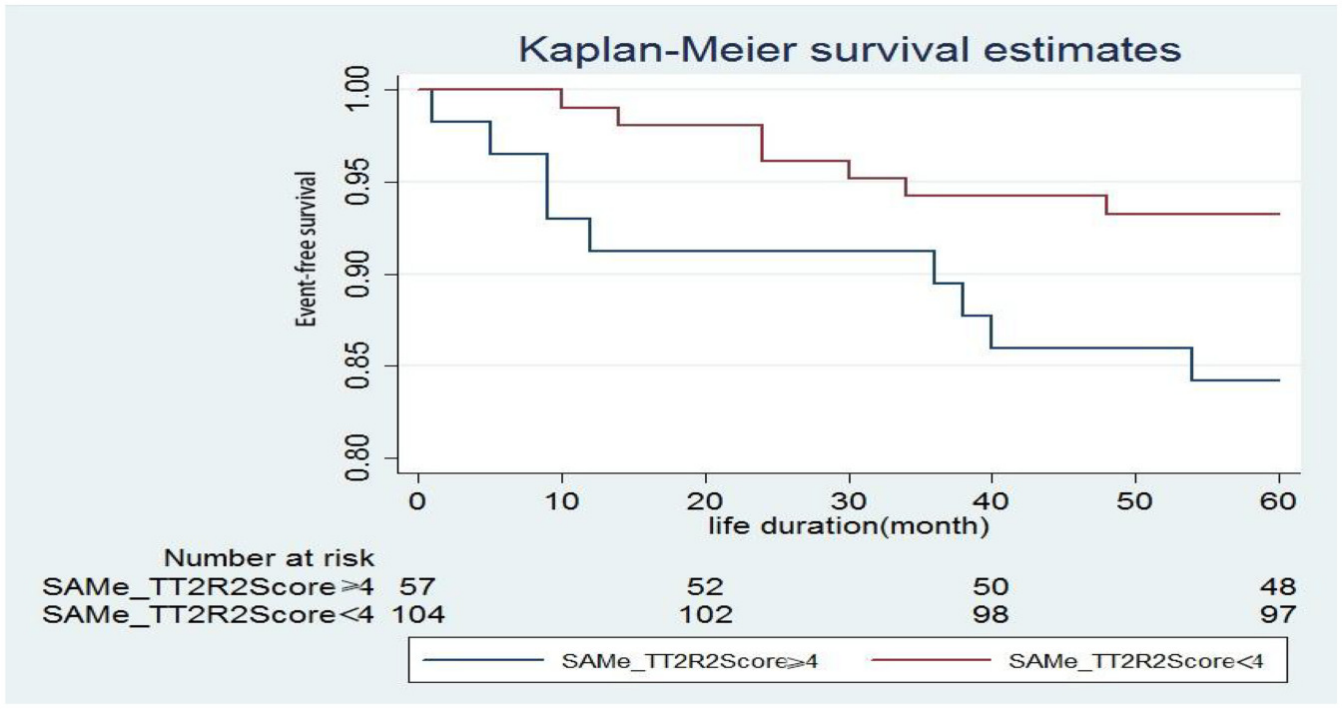
Event-free survival curve according to the points obtained in the SAMe-TT_2_R_2_ score (*p* = 0.224)

Patients were divided into 2 groups according to SAMe-TT2R2score ≥4 and SAMe-TT2R2score ≤4. The *P* value between the two groups was 0.224 calculated by chi-square test, and there was no statistical difference. Figure 2 shows the event-free survival curve.

## Discussion

In this study, we show for the first time that after mechanical mitral valve replacement, the SAME-TT_2_R_2_ model can effectively predict the level of TTR during the course of oral warfarin anticoagulation therapy. Additionally, the SAMe-TT_2_R_2_ score ≥4 can be used to predict TTR <65%.

Mitral valve replacement is a common surgical treatment for heart mitral valve disease. The existing artificial valves are divided into biological and mechanical valves. Mechanical valves have a higher usage rate among the youth due to biological valves having a certain probability of damage (13). At present, the most widely used anticoagulant after mechanical valve replacement is warfarin (14). The safety window of warfarin's effective and toxic dose is very narrow. Oral warfarin treatment requires continuous monitoring of the INR (15). Insufficient or excessive anticoagulation strength leads to bleeding and thromboembolic complications, which can be life-threatening in severe cases. After replacement, the incidence of thromboembolic events ranges from 0.4% to 1.6% per year and increases to 2.5% during the first postoperative month, despite anticoagulation therapy. Therefore, the anticoagulation quality seriously affects patients' quality of life after mechanical valve surgery (16). TTR is used as a standard to measure the quality of anticoagulation, and TTR >65%–70% is generally considered ideal for anticoagulation quality, and these patients can often benefit from it (17).

It will be extremely beneficial for clinicians to create a simple and effective tool to predict whether the patients taking warfarin orally will achieve higher TTR. Based on this, Apostolakis et al. proposed the SAME-TT_2_R_2_ model, which aimed to identify patients with Atrial Fibrillation (AF) who need additional intervention during oral warfarin anticoagulation to achieve an acceptable level of warfarin control. When the score was 0–1, the target TTR could be reached, while the score ≥2 points meant poor TTR. Certain studies have confirmed that this model can effectively predict TTR and anticoagulant adverse events. Chan et al. confirmed that in Chinese patients with AF, the SAMe-TT_2_R_2_ score had a good correlation with TTR. When the SAMe-TT_2_R_2_ score was >2 points, TTR had high sensitivity and negative predictive value, and the risk of ischemic stroke gradually increased with the increase of the SAMe-TT_2_R_2_ score (18). Fernando Pivatto Júnior et al. found that the SAMe-TT_2_R_2_ score can effectively predict the TTR level of oral warfarin in patients with AF. For patients with a higher SAMe-TT_2_R_2_ score (≥2 points), the anticoagulant effect of warfarin was poor, while patients with low-risk (0–1 points) responded better to warfarin (19). Rungroj et al. studied 1,669 patients in 22 centers across Thailand and found that the SAMe-TT_2_R_2_ score was the only independent predictor of sub-optimal TTR among NVAF patients treated with warfarin (20). However, some studies have contradicted these findings. A systematic review and simulation meta-analysis conducted by Miert et al. pointed out that although the SAMe-TT_2_R_2_ score could predict low TTR, its effect was limited and not very useful clinically (21).

Few people paid attention to the quality of anticoagulation of patients after mechanical mitral valve replacement, especially in Asia. In China, except for a few large-scale cardiac treatment centers, most hospitals do not have dedicated anticoagulation clinics and anticoagulation follow-up databases (22). Quickly and effectively identifying the patients with low TTR after mitral valve replacement is critical in busy clinical work. Our study observes for the first time that the proportion of TTR ≥65% after mitral valve replacement only accounted for 29.5%, indicative of an overall low TTR in this patient group. The situation is similar in the entire Asian region (20, 23). Simultaneously, we found that the SAMe-TT_2_R_2_ score is significantly correlated with suboptimal INR control. SAME-TT_2_R_2_ score ≥4 points can predict patients with TTR <65% after mitral valve replacement. The six simple clinical variables in the SAME-TT_2_R_2_ model can help determine which patients may have poor anticoagulation quality after mitral valve replacement. Clinicians can more intuitively and easily identify these patients to carry out more effective interventions. From the event-free survival curve, the overall survival rate of patients with SAME-TT_2_R_2_ score <4 points was better than that of patients with SAME-TT_2_R_2_ score ≥4 points. This difference was not statistically significant, which may be due to the small sample size.

Although multiple studies have demonstrated that non-valvular AF patients with SAMe-TT_2_R_2_ scores >2 in Western populations may exhibit poorer anticoagulation outcomes with warfarin (18–20), limited research has evaluated the applicability of this scoring system in Asian populations—particularly for assessing anticoagulation quality after mechanical valve replacement. Notably, due to ethnic differences, the minimum baseline score in our cohort was 2 (with no patients scoring 0 or 1). Thus, acquiring just 2 additional points from other SAMe-TT_2_R_2_ variables already carries significant clinical implications. The model's predictive value varies across populations due to divergent lifestyles and risk perceptions. For instance, while younger age is considered a risk factor in Western cohorts, our clinical experience suggests that younger Asian patients demonstrate better understanding of post-operative anticoagulation importance and superior medication adherence.

As a single-center study, our findings lack direct comparison between Western and Asian populations, which may introduce bias in score interpretation. Furthermore, the SAMe-TT_2_R_2_ model does not account for all potential factors influencing time in TTR, such as alcohol consumption, genetic predispositions, or the prevalent use of traditional herbal medicines among Asians—limitations that warrant future investigation.

Nevertheless, this study provides preliminary evidence that the SAMe-TT_2_R_2_ model effectively predicts TTR levels in Asian patients receiving warfarin after mechanical mitral valve replacement. Its simplicity facilitates rapid identification of high-risk patients requiring intensified monitoring, early intervention, and targeted education to mitigate anticoagulation-related complications.

## Limitations

This study is affected by the small sample size of a single-center, and future data will continue to be collected. The SAME-TT_2_R_2_ model does not include all potential factors that may affect TTR, such as drinking and genetic predisposition, which will be continuously improved in future studies.

## Conclusion

After mechanical mitral valve replacement, the SAME-TT_2_R_2_ model can effectively predict TTR levels during the course of using oral warfarin anticoagulation. Also, the SAMe-TT_2_R_2_ score ≥4 can predict TTR <65%.

## Data Availability

The original contributions presented in the study are included in the article/Supplementary Material, further inquiries can be directed to the corresponding author.
